# Brain PET motion correction using 3D face-shape model: the first clinical study

**DOI:** 10.1007/s12149-022-01774-0

**Published:** 2022-07-19

**Authors:** Yuma Iwao, Go Akamatsu, Hideaki Tashima, Miwako Takahashi, Taiga Yamaya

**Affiliations:** Department of Advanced Nuclear Medicine Sciences, Institute for Quantum Medical Science, National Institutes for Quantum Science and Technology (QST), 4-9-1 Anagawa, Inage-ku, Chiba, 263-8555 Japan

**Keywords:** Brain PET, Motion correction, Range-sensing camera, Kinect, 3D face-shape model

## Abstract

**Objective:**

Head motions during brain PET scan cause degradation of brain images, but head fixation or external-maker attachment become burdensome on patients. Therefore, we have developed a motion correction method that uses a 3D face-shape model generated by a range-sensing camera (Kinect) and by CT images. We have successfully corrected the PET images of a moving mannequin-head phantom containing radioactivity. Here, we conducted a volunteer study to verify the effectiveness of our method for clinical data.

**Methods:**

Eight healthy men volunteers aged 22–45 years underwent a 10-min head-fixed PET scan as a standard of truth in this study, which was started 45 min after ^18^F-fluorodeoxyglucose (285 ± 23 MBq) injection, and followed by a 15-min head-moving PET scan with the developed Kinect based motion-tracking system. First, selecting a motion-less period of the head-moving PET scan provided a reference PET image. Second, CT images separately obtained on the same day were registered to the reference PET image, and create a 3D face-shape model, then, to which Kinect-based 3D face-shape model matched. This matching parameter was used for spatial calibration between the Kinect and the PET system. This calibration parameter and the motion-tracking of the 3D face shape by Kinect comprised our motion correction method. The head-moving PET with motion correction was compared with the head-fixed PET images visually and by standard uptake value ratios (SUVRs) in the seven volume-of-interest regions. To confirm the spatial calibration accuracy, a test–retest experiment was performed by repeating the head-moving PET with motion correction twice where the volunteer’s pose and the sensor’s position were different.

**Results:**

No difference was identified visually and statistically in SUVRs between the head-moving PET images with motion correction and the head-fixed PET images. One of the small nuclei, the inferior colliculus, was identified in the head-fixed PET images and in the head-moving PET images with motion correction, but not in those without motion correction. In the test–retest experiment, the SUVRs were well correlated (determinant coefficient, *r*^2^ = 0.995).

**Conclusion:**

Our motion correction method provided good accuracy for the volunteer data which suggested it is useable in clinical settings.

**Supplementary Information:**

The online version contains supplementary material available at 10.1007/s12149-022-01774-0.

## Introduction

Positron emission tomography (PET) radiopharmaceuticals targeting amyloid-β or tau protein are becoming more available [1–4], and the number of people with cognitive impairments is estimated to be increasing [5–7], therefore the brain PET imaging should be more helpful for these individuals. To obtain high-quality brain images, patients are required to keep their head in a fixed position during PET scanning, which usually lasts at least 10 min; however, this is burdensome for patients. If the PET system is equipped with a motion correction method, the burden can be reduced.

One of the important elements of motion correction is motion tracking. Tracking methods have been investigated by many researchers and fall into two categories; One is “data-driven methods”, in which head motions are estimated by the list-mode data; [8, 9]. This method can be implemented as software without external devices to detect the head motion, but unfortunately, gradual slow movement in one direction is sometimes difficult to be identified by data-driven methods, or when using the neuronal network, a sufficient training data set is needed. The second group methods directly measure the head motion using external markers and their sensor, in which the motion parameters are applied to the list-mode data to align them to the initial position. As external sensors, stereo cameras or time-of-flight type range sensors are often used [10–15]. In this second group, a spatial calibration unifying the two coordinate systems of the external sensor and PET system is critical because it affects the accuracy of the reconstructed images. However, there are some problems in the spatial calibration; external sensors cannot be moved once the sensor is set to track the markers, and strict handling of the external sensor and markers becomes another burden for patients as well as medical staff in clinical practice.

To achieve simple and accurate head motion correction, we focused on the complexity of a face shape. 3D face-shape model can be generated by a range-sensing camera (Kinect; Microsoft Corporation, WA, US) and also by CT images. In our method, the facial surface shape is used as the markers and also used for the spatial calibration between Kinect and PET, and Kinect is simply placed in front of the subject during the PET scan. We have successfully corrected images of a moving mannequin-head containing 3-mm diameter rods filled with ^22^Na [16], where we used our developed brain-dedicated PET with 4-mm full width at half maximum (FWHM) spatial resolution and with 245 ps time-of-flight resolution [17]. In the experiment, the mannequin was held by hand and given random motions like human movement, but the movement axis is more complicated in humans because the head can be moved by the articulations and many muscles, which are not equipped with the mannequin. Furthermore, face complexity varies in volunteers. Here, we verified the feasibility and the accuracy of our method in a study with human volunteers.

## Materials and methods

### Participants and data acquisition

**Fig. 1:**
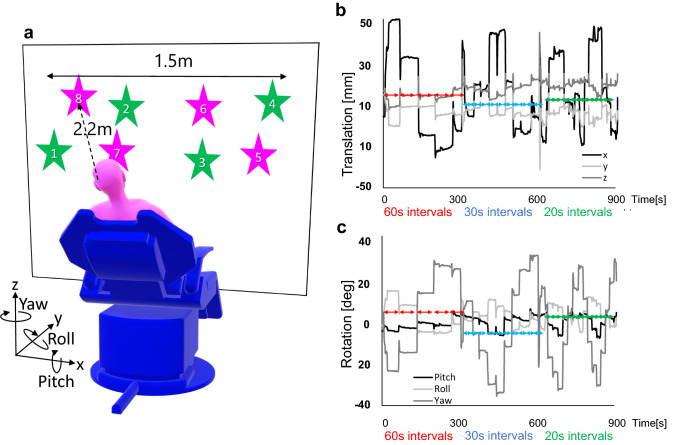
An illustration of markers on the wall for participants to move their head by seeing the marks one after another (**a**). A representative diagram of the motion amount, in which the amounts of translation in each direction, x, y, and z axis (**b**), and the rotation amounts in each direction (**c**). The head-moving PET scans were composed of three 5-min sets of 60, 30 and 20 s intervals during which participants were requested to move their head

A set of data of individual brain PET, CT, MRI images, and motion-tracking data were obtained from eight healthy men volunteers aged 22–45 years, who had no medical history of brain injuries, psychiatric disorders, or abnormal findings on MRI. ^18^F-fluorodeoxyglucose (FDG) of 285 ± 23 MBq was administered after fasting for longer than 6 h. At 45 min after the injection, the first PET scan was performed for 10 min with the forehead and chin fixed with dedicated bands, called head-fixed PET and used as a standard of truth in this study. Each participant underwent the next PET scan for 15 min using our motion-tracking system, in which all the participants were instructed to move their head to see the markers on the wall in front of them in ascending order from marker No.1 to No.8, repeatedly (Fig. 1a). The timing of the head movement from one marker to another was about 60 s for the first 5-min interval, 30 s for the next 5-min interval, and 20 s for the last 5-min interval. Each 5-min interval was cued by us, but the timing of the head movements within that interval depended on each participant counting the seconds in their mind. A representative diagram of the motion amount is shown in Fig. 1b, c. This head-moving PET scan was done in list-mode data acquisition. CT image was obtained separately on the same day using a PET-CT scanner (Discovery MI; GE Healthcare, Milwaukee, WI, USA) with acquisition parameters set at 200 mA and 120 kV. The voxel size was 0.5469 × 0.5469 mm, slice thickness was 3.75 mm, and matrix size was 512 × 512 pixels.

This prospective study was performed in accordance with the Declaration of Helsinki and was approved by the institutional review board of our institute’s hospital. Informed consent was obtained from all participants.

### Spatial calibration between the coordinate systems of PET and Kinect

**Fig. 2:**
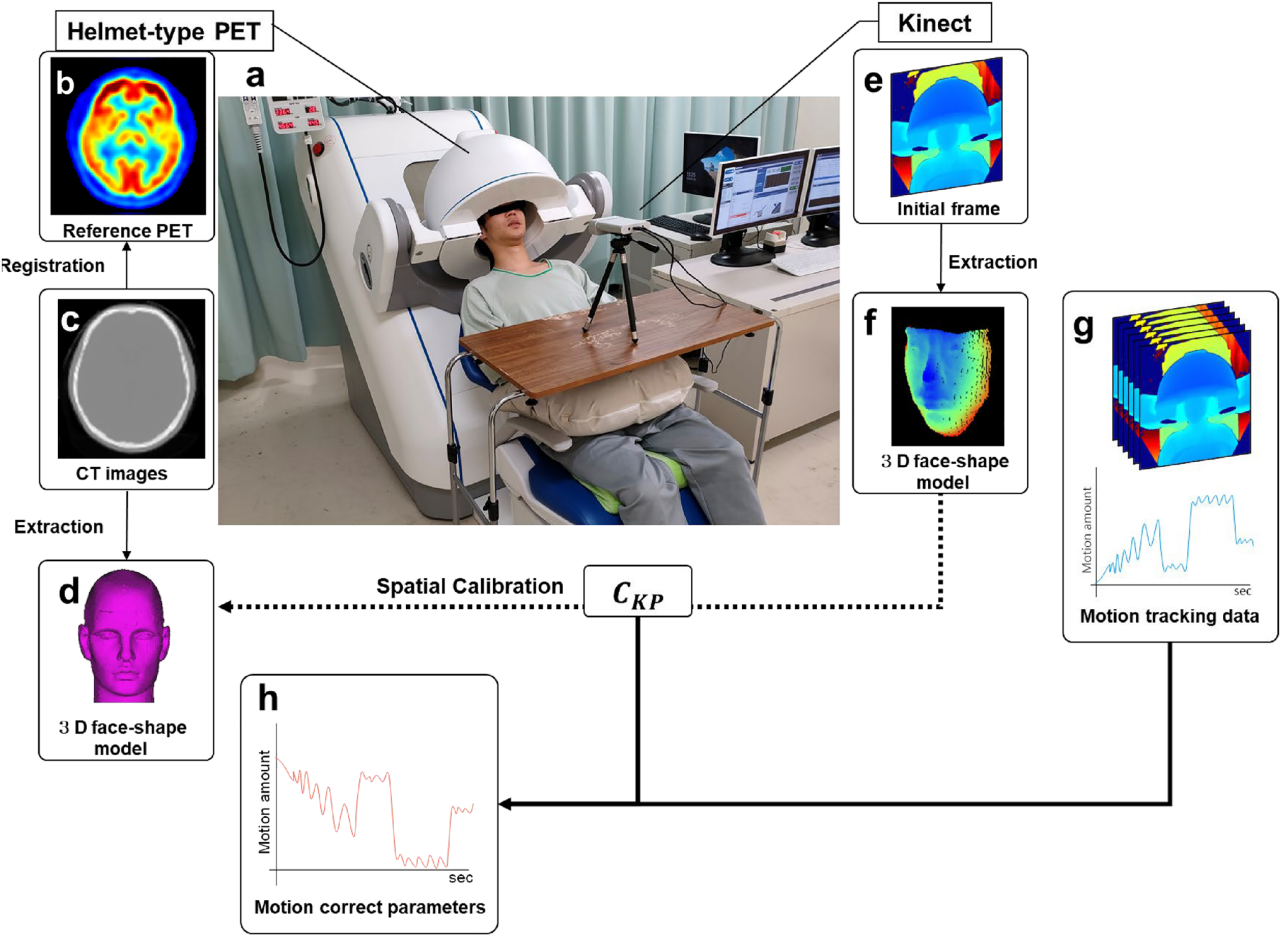
An overview of the spatial calibration and motion correction. A photo taken during the head-moving PET scan with a motion tracking system (Kinect) (**a**). The participant was reclining with his head inside the helmet-type PET without any head fixation. A reference PET (**b**) is obtained from a motion-less period from the PET list-mode data, to which the CT image was registered (**c**), and generates a 3D face-shape model (**d**). The initial frame from Kinect data determines the face position (**e**), and generates a 3D face-shape model (**f**) by contouring the face area on the Kinect display. A representative Kinect display is shown in (**e**). These two face-shape models matching provides the calibration parameters $C_{{{\text{KP}}}}$, which become the parameter to calibrate the coordinate systems of Kinect and PET. The amount of motion is calculated by matching each frame of the motion tracking to the initial position (**g**), and calculated motion correctio parameters (**h**)

A flowchart of motion correction process and an overview of the setting of our motion-tracking system and the brain-dedicated PET are shown in Fig. 2a. Details of our motion-tracking system using Kinect (Microsoft Corporation, WA, US) were described previously [16, 18]. Briefly, Kinect is a range-sensing camera by measuring the time difference between the infrared emission and its return to the sensor. We synchronized Kinect and PET with the frame rate of 10 frames per second and have proved the tracking error to be less than 1 mm and less than 1 degree [18]. First, we selected motion-less periods of about 20 s from the PET list-mode data concerning the motion tracking data, and we reconstructed image using the selected list-mode events without attenuation correction, which were used as the reference PET image for motion correction (Fig. 2b). Second, we registered the CT image to the reference PET image by optimizing the six parameters necessary for rigid alignment using the Nelder-Mead method [19] (Fig. 2c), and created a 3D face-shape model (Fig. 2d). Here, we defined the 3D face-shape model as 3-dimensional information of the face surface of individuals’, which is created by CT and is also by Kinect data. Kinect-based 3D face-shape model, composed of a set of the points of the face surface, was created by contouring the face area, a “face region-of-interest (ROI),” with Kinect viewer, which is 2D display but including 3D information. Third, a reference face position was created by placing a face ROI on the initial frame of the Kinect data (Fig. 2e), and created a 3D face-shape model (Fig. 2f). As these two 3D face-shape models by CT and by Kinect represent the same object in real space, they can be properly superimposed by iterative closest point (ICP) algorithm [20]. In ICP algorithm, pairs of nearest neighbors of the reference and target models area extracted. Until the distance between the pairs is converged to a minimum, the coordinate transformation is repeated. Here, the homothetic transformation matrix ${C}_{KP}$ expresses the amount of movement to transform the Kinect coordinate system to the PET coordinate system. During the motion tracking, all frames were matched with the reference face position using ICP algorithm and then, the amounts of the motion were calculated (Fig. 2g). The $C_{{{\text{KP}}}}$ and amounts of movement provide motion correction parameters (Fig. 2h).

### Image reconstruction with motion correction

The image reconstruction was based on the motion-compensation ordered subset expectation maximization (OSEM) list-mode algorithm for resolution-recovery/reconstruction (MOLAR) method as proposed in the literature [21, 22]. The acceleration method was constructed using graphics processing unit (GPU) operations. The MOLAR method algorithm we used in this study included scatter correction by single-scatter simulation method based on CT images, which is proposed by C.C. Watson [23], and parameters are used according to the previous reports [21, 22]. PET images were smoothed by a 3D Gaussian filter of 4 mm in FWHM with a voxel size of 2.0 × 2.0 × 2.0 mm^3^ and matrix size of 140 × 140 × 112 pixels.

The motion in each frame was expressed as a transformation matrix ${\mathbf{\Im }}$ in a rigid body by the following equation. ${\mathbf{\Im }}$ was calculated as a parameter to match the frame data to the reference model.

1 $$\Im = \left( {\begin{array}{*{20}c} R & s \\ 0 & 1 \\ \end{array} } \right) = \left( {\begin{array}{*{20}c} {r_{11} } & {r_{12} } & {r_{13} } & {s_{x} } \\ {r_{21} } & {r_{22} } & {r_{23} } & {s_{y} } \\ {r_{31} } & {r_{32} } & {r_{33} } & {s_{z} } \\ 0 & 0 & 0 & 1 \\ \end{array} } \right),$$

Here $\user2{ }R$ is a $3 \times 3$ rotation matrix ($r_{11} , \cdots ,r_{33}$ are the components) and ${\varvec{s}}$ is a $3 \times 1$ translation vector ($s_{x} ,s_{y} ,s_{z}$ are translation amounts in the $x,y,z$ axes). The motion data from Kinect, $\Im_{{\text{K}}}$ are converted into the invert motion data $\Im_{{\text{P}}}$ in the PET coordinate system by the following formula,

2 $$\Im_{{\text{P}}} = C_{{{\text{KP}}}}^{ - 1} \Im_{{{\text{K}} }}^{ - 1} C_{{{\text{KP}}}}$$

### Quantitative evaluation of images

The head-moving PET images with motion correction were compared with head-fixed PET images as the mean value of the standard uptake value ratio (SUVR) using a template of anatomical 3D volumes of interest (VOIs) [24]. We used PMOD Ver. 3.7 (PMOD Technologies, Switzerland) for the VOI analysis. PET images were co-registered to individual 3D T1-weighted MRI. The VOI template was applied to individual PET images using inverse transformation parameters of MRI-based spatial normalization. Eight VOIs were selected as: frontal, medial temporal, lateral temporal, parietal, occipital cortex, posterior cingulate and precuneus, striatum, and cerebellar. SUVRs were calculated using the SUV of each VOI divided by the SUV of the cerebellar VOI.

In addition, the brain-dedicated PET visualizes the inferior colliculus in the midbrain, which is composed of two lobes bilaterally with a size of approximately 6 mm in diameter [25], therefore we obtained a profile curve of voxel values on the inferior colliculus in the PET images with the voxel size of 2.0 × 2.0 × 2.0 mm^3^ for each participant and calculated the peak-to-valley ratio.

### Test–retest experiment of the spatial calibration

To demonstrate the accuracy of the spatial calibration method, we repeated 15-min head-moving PET scans twice with the same moving pattern for one participant, called calibration 1 and calibration 2. Between the two scans, we asked the participant to leave the PET to take a 5-min break before making the second PET scan. During the break, the participant could stand and stretch. After the participant sat in the PET seat again, the Kinect system was placed in front of him, not precisely at the same place as for the first scan. Therefore, the participant’s posture and Kinect placement differed between calibration 1 and 2. Each of these two head-moving PETs was reconstructed with motion correction and was compared regarding SUVR for the same regions as described above.

### Statistics

Wilcoxon signed-rank test was used for the comparisons between the head-moving PET with motion correction and the head-fixed PET in SUVRs of representative seven VOIs and in peak-to-valley ratio, and used for the comparison between calibration 1 and 2 in SUVR of the seven VOIs. Statistical significance was set at *p* < 0.05.

## Results

**Fig. 3:**
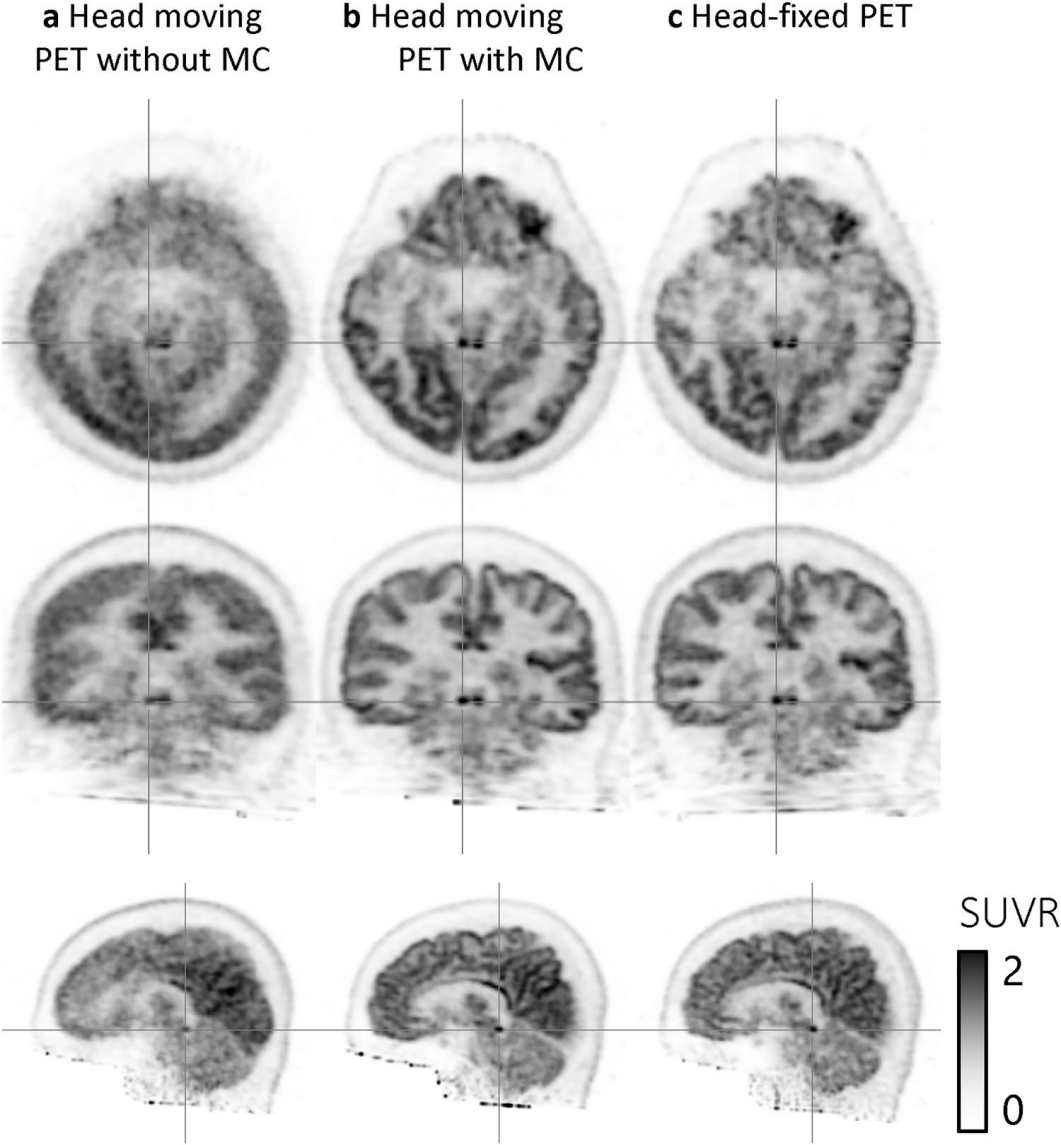
Representative images from participant 3: the head-moving PET without motion correction (MC) (**a**), with MC (**b**) and the head-fixed PET images (**c**). Axial, coronal, and sagittal views are shown in order of top to bottom row images

The amounts of the movement were varied among participants; the maximum 56 mm, average 28.5 mm, and minimum 12 in translation, and 40 degrees, 27.3 degrees, and 20 degrees in rotation, respectively. Representative images of head-moving PET without and with motion correction, and head-fixed PET are shown in Fig. 3. The head-moving PET with motion correction and head-fixed PET images were visually almost the same. One of the small nuclei, the inferior colliculus in the midbrain, was clearly visualized in the head-moving PET with motion correction and head-fixed PET images, but not in the head-moving PET without motion correction.

### Quantitative evaluation of images

**Fig. 4:**
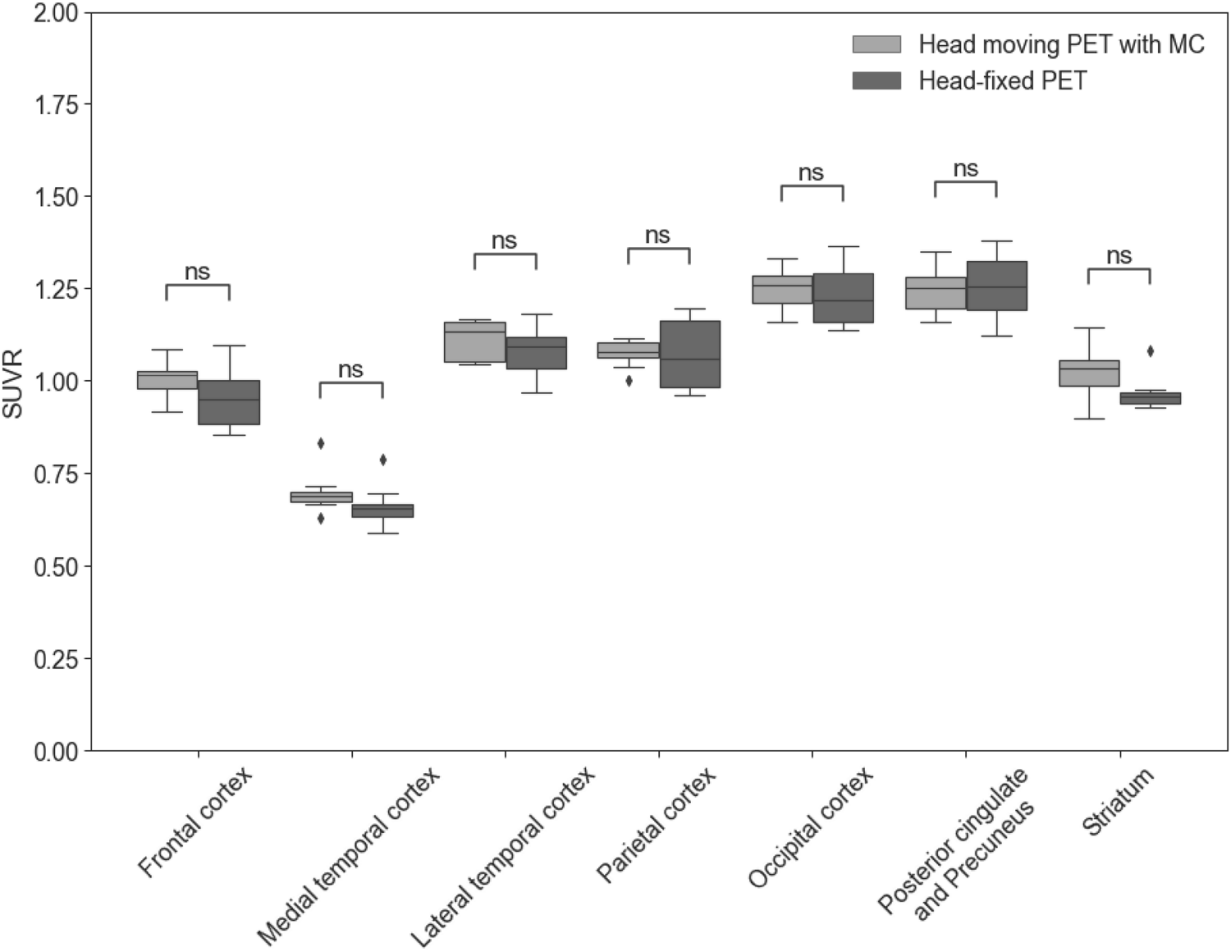
Box-and-whisker plots of SUVRs in eight VOIs. There was no statistical difference in SUVRs between the two PET images. Central horizontal line, median value; horizontal ends of the box, upper and lower quartiles; the bar ends, maximum and minimum values; dots show the outliers

A box-and-whisker plot of SUVRs between the head-moving PET with motion correction and the head-fixed PET images is shown in Fig. 4. There was no statistical difference among the seven VOIs (frontal, *p* = 0.75; medial temporal, *p* = 0.11; lateral temporal, *p* = 0.38; parietal, *p* = 1.00; occipital cortex, *p* = 1.00; posterior cingulate and precuneus, *p* = 1.00; striatum, *p* = 0.27).

**Fig. 5:**
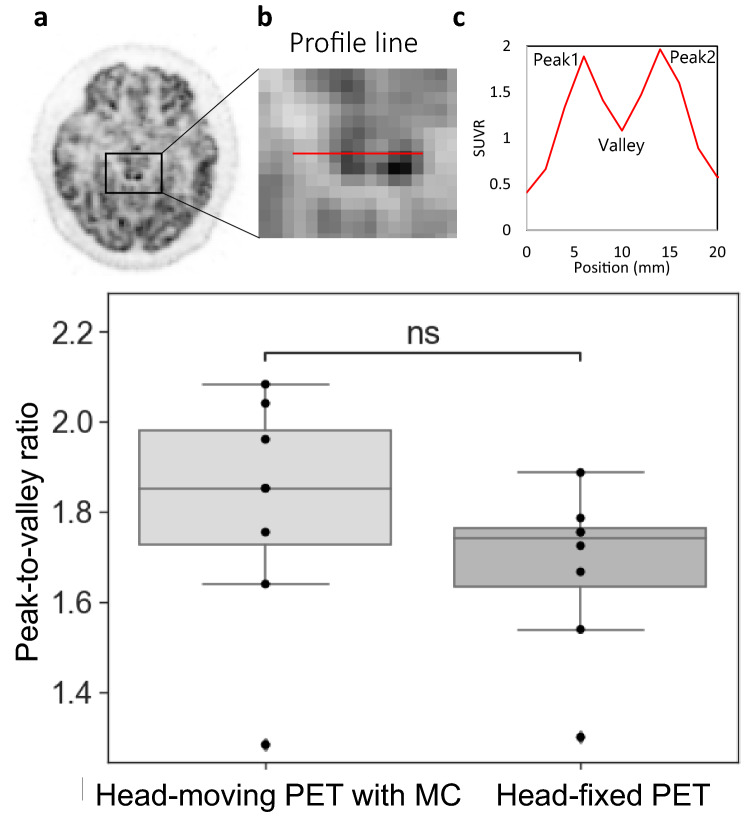
Comparison of the peak-to-valley ratio in the profile line on the inferior colliculus: target slice (**a**), profile line setting (**b**), example of profile curve (**c**). Peak-to-valley ratios were calculated by the average of peak1 and peak2 divided by the valley and compared between head-moving PET with motion correction (MC) and head-fixed PET (**d**)

An example of the profile line through the inferior colliculus obtained from the head-moving PET with motion correction and the head-fixed PET is shown in Fig. 5a–c. The peak-to-valley ratio is plotted in Fig. 5d; a higher ratio suggests higher contrast. There was no statistical significance (*p* = 0.47), but the ratio tended to be higher in the head-moving PET with motion correction than in the head-fixed PET.

### Test–retest experiment of the spatial calibration method

**Fig. 6:**
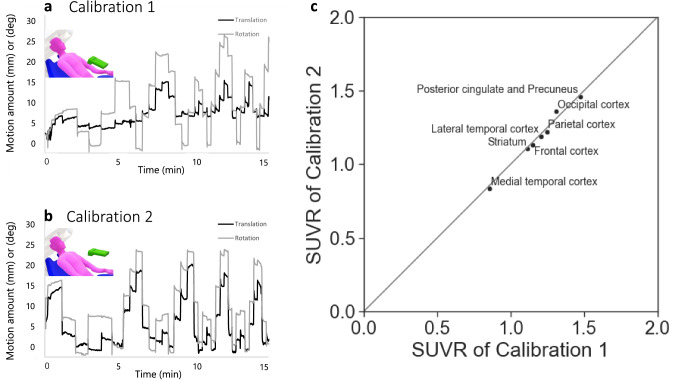
Diagrams of the motion amount of calibration 1 (**a)** and 2 (**b)** are sequentially obtained from the same volunteer, but there are differences in the volunteer’s pose and the sensor’s position. A comparison of SUVR in eight regions between calibrations 1 and 2 shows well correlation (determinant coefficient, *r*^2^ = 0.995; linear approximation equation, *y* = 1.033*x*) (**c**)

The motion amount data during the two PET scans for one participant are shown in Fig. 6a, b. SUVRs calculated from two PET scans with motion correction were well correlated (determinant coefficient; *r*^2^ = 0.995, Fig. 6c). Furthermore, no statistically significant differences in SUVR between calibrations 1 and 2 were identified.

## Discussion

We evaluated the developed motion correction method in the volunteer study. Compared to the head-fixed PET images, no degradation was visually identified on the head-moving PET with motion correction, and no statistical difference was found between them by VOI analysis. The contrast in images for the small nuclei, the inferior colliculus, was evaluated using the peak-to-valley ratio obtained by the profile curve and tended to be slightly better in images from the head-moving PET with motion correction than in the head-fixed PET images. We think that the reason why the contrast slightly increased was due to the “Wobbling effect [26].” When the scanner or object shifts less than the width of detector crystals, the spatial sampling rate of line-of-interest (LOR) is increased because LORs can be also obtained from between the LORs of the fixed scanner and object (Supplementary Fig. 1).

Motion correction methods with simple marker attachment or without any markers are preferred not only for patients but also in various clinical studies [10, 14, 15], for example, PET scan taken during interventions such as cigarette smoking. Olesen et al. successfully corrected the head movement up to 18 mm in a volunteer smoking study during a PET scan with a CCD camera for motion tracking [10]. In our previous study using a mannequin head, the amount of the motions up to 20 mm in translation and 30 degrees in the rotation were corrected, demonstrating a 3-mm diameter rod clearly separated with our motion correction [16]. In this study, the amount of the motions was up to 50 mm in translation for each direction and 40 degrees in the rotation of each axis were observed, and successfully corrected. There are two possible reasons why our method was able to correct such a large amount of movement. The first one is the face ROI used for the motion tracking, where the ROI was limited to a relatively small are, including the eyes and nose, rather than the entire face because the other facial areas except the eyes and nose area have few geometrical features, therefore reducing the accuracy through ICP matching [18]. The second point is pre-processing which transforms each frame into the previous frame before the matching processes between the reference 3D face-shape model and each frame. This pre-processing allows the initial value of the matching calculation to be as close to the reference as possible, improving the matching accuracy [18]. Compared to the data-driven method, in which slow motion is difficult to detect, our method directly measures motion at approximately 10 frame per second with the accuracy approximately 1 mm and 1 degree in a mannequin experiment. We believe that the larger motion that can be corrected, the more useful the method for many patients.

The test–retest experiment concerning the spatial calibration method showed that the two scans were identical after the motion correction, although the participant’s pose and the sensor’s position were different. Most motion corrections using external sensors require rigorous handling of the sensors to fix them at the first position. In our method, the spatial calibration between the different coordinate systems was performed using an individual’s face-shape model created by Kinect and by CT, and PET images were easily aligned to CT images. Therefore, our method is not limited to the brain-dedicated PET we developed, but, to be used for other current PET systems, we need to confirm that the eyes and nose can be monitored by Kinect which are synchronized with the PET system, to acquire PET list-mode data can be acquired, and the program for image reconstruction based on MOLAR method needs to be implemented.

The reference PET image in this study was the basis for the spatial calibration between PET and Kinect coordinate systems and the calculation of the head movement during scan. In this study, the reference PET images were generated by manually selecting motion-less periods for about 20 s from the list-mode PET data, which may cause inter-operator variation. To automatically generate reference PET images, it is necessary to set a threshold value for the amount of movement in the motion tracking data and select the motion-less time periods. In addition, if the movements are so frequent that a continuous 20-s static interval cannot be selected from the motion tracking data, it is possible to select a total of 20 s that are discontinuous but almost identical in posing.

The limit of the movement amount that our method has corrected is up to 45 degrees in the rotation because when the rotation is larger than 45 degrees the eyes and nose is hidden from the view of Kinect [18]. As for parallel translation, within the field of view of the PET system the amount of movement is not limited. Despite this range of limitations, our method will be helpful for patients with cognitive impairment as well as healthy volunteers. Another limitation of this study was that the true amount of the volunteers’ movement is unknown, therefore there is no way to confirm the accuracy of the motion estimation with our method in each frame, but no large outliers or interruptions of the tracking process were observed.

## Conclusion

The developed motion correction method using 3D face-shape model created by a range-sensing camera (Kinect) and by CT was successfully corrected brain PET images with the amount of motion up to 50 mm in translation and 40 degrees in the rotation of each axis.

## Supplementary Information

Below is the link to the electronic supplementary material.

Supplementary file1 (DOCX 58 KB)
