# Supplementary material for: Brain PET motion correction using 3D face-shape model: the first clinical study
Source: Ann Nucl Med. 2022 Jul 19;36(10):904–12. doi: 10.1007/s12149-022-01774-0 (PMC9515015; doi:10.1007/s12149-022-01774-0)
Supplement: Supplementary file 1 — Supplementary file1 (DOCX 58 KB) [file 12149_2022_1774_MOESM1_ESM.docx]

Brain PET motion correction using 3D face-shape model: the first clinical study

*Annals of Nuclear Medicine*

Yuma Iwao, PhD,^1^ Go Akamatsu, PhD,^1^ Hideaki Tashima, PhD, ^1^ Miwako Takahashi, MD, PhD, ^1^ Taiga Yamaya, PhD^1^

1 Department of Advanced Nuclear Medicine Sciences, Institute for Quantum Medical Science, National Institutes for Quantum Science and Technology (QST), Chiba, Japan.

Corresponding author:　Miwako Takahashi　(E-mail: takahashi.miwako@qst.go.jp)


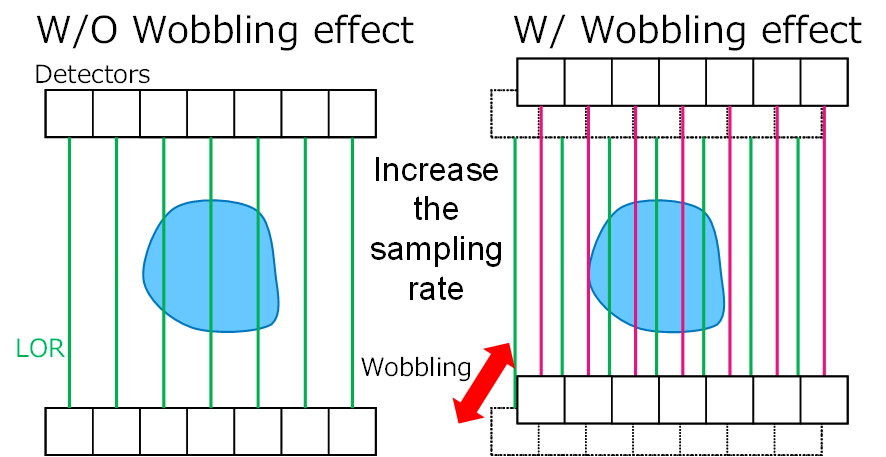


**Supplementary Figure 1:** Schematic illustration of the Wobbling effect.

Line-of-interest (LOR) was represented in green when the scanner and object are fixed. When the scanner and object shift less than the width of detector crystals, spatial sampling rate of line-of-interest (LOR) is increased because LORs (red line) can be also obtained from between the LORs of the fixed scanner and object.
